# Heme Oxygenase-1, a Key Enzyme for the Cytoprotective Actions of Halophenols by Upregulating Nrf2 Expression via Activating Erk1/2 and PI3K/Akt in EA.hy926 Cells

**DOI:** 10.1155/2017/7028478

**Published:** 2017-06-14

**Authors:** Xiu E. Feng, Tai Gang Liang, Jie Gao, De Peng Kong, Rui Ge, Qing Shan Li

**Affiliations:** ^1^School of Pharmaceutical Science, Shanxi Medical University, Taiyuan 030001, China; ^2^Shanxi University of Traditional Chinese Medicine, Taiyuan 030024, China

## Abstract

Increasing evidence has demonstrated that heme oxygenase-1 (HO-1) is a key enzyme triggered by cellular stress, exhibiting cytoprotective, antioxidant, and anti-inflammatory abilities. Previously, we prepared a series of novel active halophenols possessing strong antioxidant activities in vitro and in vivo. In the present study, we demonstrated that these halophenols exhibited significant protective effects against H_2_O_2_-induced injury in EA.hy926 cells by inhibition of apoptosis and ROS and TNF-*α* production, as well as induction of the upregulation of HO-1, the magnitude of which correlated with their cytoprotective actions. Further experiments which aimed to determine the mechanistic basis of these actions indicated that the halophenols induced the activation of Nrf2, Erk1/2, and PI3K/Akt without obvious effects on the phosphorylation of p38, JNK, or the expression of PKC-*δ*. This was validated with the use of PD98059 and Wortmannin, specific inhibitors of Erk1/2 and PI3K, respectively. Overall, our study is the first to demonstrate that the cytoprotective actions of halophenols involve their antiapoptotic, antioxidant, and anti-inflammatory abilities, which are mediated by the upregulation of Nrf2-dependent HO-1 expression and reductions in ROS and TNF-*α* generation via the activation of Erk1/2 and PI3K/Akt in EA.hy926 cells. HO-1 may thus be an important potential target for further research into the cytoprotective actions of halophenols.

## 1. Introduction

**Figure 1:**
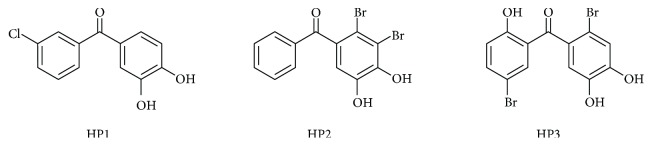
Chemical structures of halophenols HP1, HP2, and HP3.

Vascular injury, including structural and functional impairment of the endothelium, is associated with an increased risk of developing various chronic inflammatory cardiovascular diseases, such as atherosclerosis, myocardial infarction, and hypertension [1, 2]. Various halophenols naturally derived from marine algae, ascidians, and sponges usually possess 1–4 benzene rings, in which halogen atoms and hydroxyls are linked by single covalent bonds [3–7]. Recently, many novel halophenols have been studied with growing interest due to their antioxidant, antimicrobial, antithrombotic, enzyme inhibitory, and cytotoxic activities, as well as their anti-inflammatory activities and protective effects on the vascular endothelium [8–12]. These promising biological properties have encouraged the development of efficient structural optimization and investigation into the mechanistic basis of halophenols. Using rational structural optimization, we recently prepared a series of novel halophenols with benzophenone, benzylbenzene, or furan-2-yl(phenyl)methanone skeletons, and we discovered three interesting compounds, namely halophenols 1 (HP1), 2 (HP2), and 3 (HP3) (Figure 1), with significant cytoprotective activities against H_2_O_2_-induced injury in human umbilical vein endothelial cells (HUVECs) [8, 9]. Additionally, further in vivo study showed that HP3 exhibited strong antiatherosclerotic and protective effects against myocardial ischemia-reperfusion injury in rats owing to its antioxidant, anti-inflammatory, and antiapoptotic abilities [13, 14].

Prolonged exposure to oxidative stress is an important cause and risk factor of many cardiovascular diseases. Heme oxygenase-1 (HO-1) is a stress-inducible rate-limiting enzyme that catalyzes the degradation of heme to generate biliverdin, free iron, and CO and plays a vital role in the defense and repair of oxidative stress-induced damage [15, 16]. Lack of HO-1 can cause profound changes in cellular homeostasis in genetically deficient mice and humans, which is associated with susceptibility to oxidative stress [17, 18]. In addition, HO-1 is also regarded as an important protein for anti-inflammatory and antiapoptotic actions [19–21] and therefore has become a potential target for the treatment of cardiovascular diseases with high oxidative stress levels, such as atherosclerosis, myocardial ischemia-reperfusion injury, hypertension, diabetes, and obesity [22–26].

The induction of HO-1 under oxidative stress is mainly activated by the transcription factor nuclear factor erythroid 2-related factor 2 (Nrf2), which is regulated by the mitogen-activated protein kinase (MAPK), phosphoinositide 3-kinase (PI3k)/Akt, and protein kinase C (PKC) signaling pathways [27–32]. Increasing evidence has indicated that cytoprotective agents such polyphenolic antioxidants can activate Nrf2 by interacting with Keap1 to release Nrf2 from the Keap1-Nrf2 complex [33–35]. Subsequent Nrf2 translocation into the nucleus and binding to the antioxidant response element (ARE) result in the transcriptional activation of phase II antioxidant enzymes, including HO-1 [36].

Considering the beneficial properties of halophenols as well as the possible key role of HO-1, in this study, we investigated the correlation between HO-1 activation and the cytoprotective actions of halophenols in H_2_O_2_-induced EA.hy926 cells and further explored the regulatory mechanism of associated upstream signaling pathways.

## 2. Materials and Methods

### 2.1. Materials

EA.hy926 cells were obtained from the Shanghai Institutes for Biological Sciences (Shanghai, China). We obtained 3-(4,5-dimethylthiazol-2-yl)-2,5-diphenyltetrazolium bromide (MTT) and zinc protoporphyrin (ZnPP) from Sigma (St. Louis, MO, USA). TRIzol reagent was obtained from Invitrogen (Carlsbad, CA, USA). DMEM, fetal bovine serum (FBS), and cell culture reagents were obtained from HyClone (Logan, UT, USA). The reactive oxygen species (ROS) assay kit and tumor necrosis factor-α (TNF-*α*) ELISA kit were from Boster Biological Engineering Co. (Wuhan, China). Antibodies against Nrf2, HO-1, p-p38, p38, p-Erk, Erk, p-JNK, JNK, p-Akt, PKC-*δ*, and *β*-actin were purchased from Cell Signaling Technology (Danvers, MA, USA). The nuclear protein extraction kit was obtained from Beyotime Biotechnology Co. (Shanghai, China), and anti-Lamin B was from Santa Cruz Biotechnology (Santa Cruz, CA, USA). PD98059 and Wortmannin were purchased from Calbiochem (La Jolla, CA, USA). All other chemicals and reagents were of analytical grade from China.

### 2.2. Chemicals

Three halophenols, HP1, HP2, and HP3, were provided by our research group. Their purities were higher than 99.5%, as assessed by RP-HPLC on a column of Diamonsil C18 (250 mm × 4.6 mm, 5 *μ*m) with a mobile phase of 70/10/20 methanol/acetonitrile/water (adjusted to pH 3.0 with phosphoric acid), flow rate of 0.8 mL·min^−1^, column temperature of 25°C, injection volume of 20 *μ*L, and detection wavelengths of 243 nm, 254 nm, and 261 nm for HP1, HP2, and HP3, respectively (Agilent 1200, Palo Alto, CA, USA). Compounds were dissolved in dimethyl sulfoxide (DMSO), stored at −20°C, and diluted to test concentrations with culture medium immediately prior to the experiment. The final concentration of DMSO in the culture medium was less than 0.1%.

### 2.3. Cell Culture

EA.hy926 cells were cultured in DMEM supplemented with 10% heat-inactivated FBS, penicillin (100 U/mL), and streptomycin (100 U/mL) at 37°C in a humidified atmosphere containing 95% air and 5% CO_2_. All experiments were done following 6–12 passages.

### 2.4. Cell Viability Assay

An MTT assay was performed to estimate cell viability. Cells (1 × 10^4^ per well) were seeded in 96-well plates and cultured for 24 h, and then, the medium was replaced with fresh medium for different treatments. Next, 10 *μ*L of 5 mg/mL MTT in phosphate-buffered saline (PBS) was added to each well, and cells were further incubated at 37°C for 4 h. Then, the culture medium was carefully removed, and 100 *μ*L DMSO was added per well to dissolve the formed precipitate. Plates were shaken for 10 s, and OD values were measured at a wavelength of 490 nm on a Bio-Rad microplate reader (model 680, Bio-Rad, Hercules, CA, USA). Cell viability and cytoprotective rate were calculated using the following formulas, respectively: cell viability (%) = OD_Test_/OD_Control_ × 100% and cytoprotective rate (%) = (OD_Compound_ − OD_Model_)/(OD_Control_ − OD_Model_) × 100%.

### 2.5. Flow Cytometric Analysis of Apoptosis

An annexin V-fluorescein isothiocyanate (FITC)/propidium iodide (PI) double-staining kit (Life Technologies, Eugene, OR, USA) was used to detect apoptosis. After treatment with various concentrations of HP3, HP2, HP1, and/or H_2_O_2_, EA.hy926 cells were collected and washed twice with ice-cold PBS and then resuspended in binding buffer at a concentration of 1 × 10^6^ cells/mL. Then, 10 *μ*L of annexin V-FITC and 10 *μ*L of PI were added. The cells were incubated for 15 min in the dark, and analysis of apoptosis was then performed on a flow cytometer (Becton Dickinson, Franklin Lakes, NJ, USA). Early and late apoptotic cells were calculated based on annexin V positivity/PI negativity and annexin V positivity/PI positivity, respectively.

### 2.6. Determination of ROS and TNF-*α* Generation

Levels of intracellular ROS were examined using 2,7-dichlorofluorescein diacetate (DCFH-DA; Boster Biological Engineering Co.) by flow cytometric analysis. Cells were seeded on a 6-well plate. Twenty-four hours later, cells were treated with the compound for 6 h, exposed to 200 *μ*M H_2_O_2_ for 30 min, washed twice with PBS, and then harvested with trypsin. Cells were subsequently incubated with 10 *μ*M DCFH-DA for 30 min at 37°C and 5% CO_2_ and washed with PBS to remove all extracellular DCFH-DA; the fluorescence of dichlorofluorescein (DCF) was measured with flow cytometry (BD Biosciences, San Diego, CA, USA) [37].

Confluent cells in 24-well plates were pretreated with the compound for 6 h and subsequently incubated with 200 *μ*M H_2_O_2_ for 30 min. TNF-*α* released into the medium was detected using an ELISA kit according to the manufacturer's instructions. The relative ratios between the control and treatment groups were compared. This assay is able to detect concentrations as low as 1 pg/mL.

### 2.7. Preparation of Nuclear Proteins

According to the nuclear protein extraction protocol, 200 *μ*L protein extraction reagent A containing 1 mM phenylmethylsulfonyl fluoride (PMSF) was added to each 20 *μ*L of collected cell precipitation, vigorously vortexed for 5 s, and then left on ice for 15 min. Next, 10 *μ*L protein extraction reagent B was added to the mixture, vigorously vortexed for 5 s, and placed on ice for another 1 min. After that, the solution was centrifuged at 12,000 ×g for 10 min at 4°C. After removal of the supernatant, 50 *μ*L nuclear protein extraction reagent containing 1 mM PMSF was added to the nuclear precipitation, mixed, and vigorously vortexed for 15 s. The mixture was kept on ice for 30 min with periodic vortexing and was then centrifuged at 12,000 ×g for 10 min at 4°C. The supernatant was stored as the nuclear fraction for western blot analysis.

### 2.8. Western Blot Assay

After treatment, cells were washed with PBS and mixed with RIPA buffer containing 1 mM EDTA, 5 mg/mL aprotinin, 2 mg/mL leupeptin, and 1 mM PMSF, followed by centrifugation at 14,000 ×g for 15 min. Protein concentration was determined using a BCA kit (Boster Biological Engineering Co.). An equal amount of protein for each sample was resolved by performing 12% sodium dodecyl sulfate-polyacrylamide gel electrophoresis (SDS-PAGE) and was then electrophoretically transferred onto nitrocellulose (NC) membranes (Millipore, Billerica, MA, USA). The membrane was blocked using 5% skim milk, incubated overnight at 4°C with primary antibody, and then washed three times for 10 min each with Tris-buffered saline containing 0.05% Tween 20 (TBST). Next, the membrane was incubated for 1 h at room temperature with horseradish peroxidase-conjugated secondary antibody and washed three times, followed by ECL detection (Boster Biological Engineering Co.) [38]. Western blotting data were quantified by Gel-Pro Analyzer 4.0, and each assay was conducted three times independently.

### 2.9. Statistical Analysis

All results are expressed as means ± standard deviations (SD). A one-way analysis of variance with Tukey's post hoc test was performed using the Statistical Package for the Social Sciences software (SPSS 17.0 for Windows, 2010; SPSS Inc., Chicago, IL, USA). Values of *P* < 0.05 were considered to indicate statistical significance. All experiments were performed in triplicate.

## 3. Results

### 3.1. Halophenols Protect EA.hy926 Cells against H_2_O_2_-Induced Cell Death

**Figure 2:**
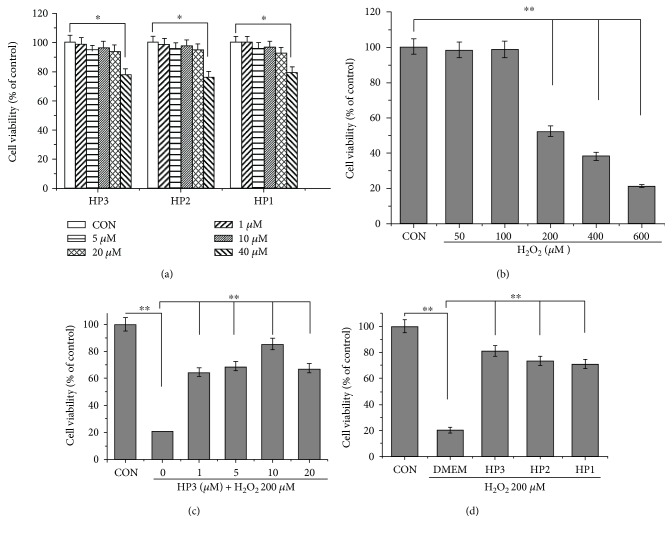
Halophenols 1 (HP1), 2 (HP2), and 3 (HP3) protect EA.hy926 cells against H_2_O_2_-induced cell death. (a) The cytotoxicities of HP1, HP2, and HP3 toward EA.hy926 cells. Cells were, respectively, incubated with three halophenols at the indicated concentrations for 24 h and their viabilities were determined by MTT. Three compounds had no influences on cell viability at concentrations between 1.0 *μ*M and 20 *μ*M for 24 h. (b) H_2_O_2_ caused a concentration-dependent reduction of cell viability in EA.hy926 cells. (c) Cytoprotection of HP3 against H_2_O_2_-induced injury in EA.hy926 cells showed a dose-dependent enhancement below 10 *μ*M. (d) Cytoprotection of 10 *μ*M HP1, HP2, and HP3 against H_2_O_2_-induced injury in EA.hy926 cells. Pretreatment of cells with 10 *μ*M HP1, HP2, or HP3 exhibited the significant cytoprotective actions with cytoprotective rates 63.3%, 65.8%, and 76.0%, respectively. ^∗^*P* < 0.05 and ^∗∗^*P* < 0.01.

The cytotoxicities of the three halophenols in the EA.hy926 cells were first examined by MTT assay at concentrations of 1, 5, 10, 20, and 40 *μ*M. As shown in Figure 2(a), the three compounds had no effects on cell viability at concentrations between 1.0 *μ*M and 20 *μ*M for 24 h. We accordingly used concentrations of halophenols up to 20 *μ*M in all additional experiments.

H_2_O_2_, a well-known cytotoxic molecule, was utilized to induce oxidative injury in EA.hy926 cells in the present study. There was a concentration-dependent decrease in cell viability after treatment with 50–600 *μ*M H_2_O_2_ for 6 h (Figure 2(b)). Compared with cell viability in the control group, a concentration of 200 *μ*M H_2_O_2_ caused about a 50% decrease in cell viability. Thus, 200 *μ*M H_2_O_2_ was selected for subsequent assays.

HP3 pretreatment at concentrations below 10 *μ*M attenuated H_2_O_2_-induced cell death in a dose-dependent manner (Figure 2(c)). Moreover, as shown in Figure 2(d), HP1, HP2, and HP3 at a concentration of 10 *μ*M each exhibited potent cytoprotective activities, with cytoprotective rates of 63.3%, 65.8%, and 76.0%, respectively.

### 3.2. Flow Cytometric Analysis of Apoptosis

**Figure 3:**
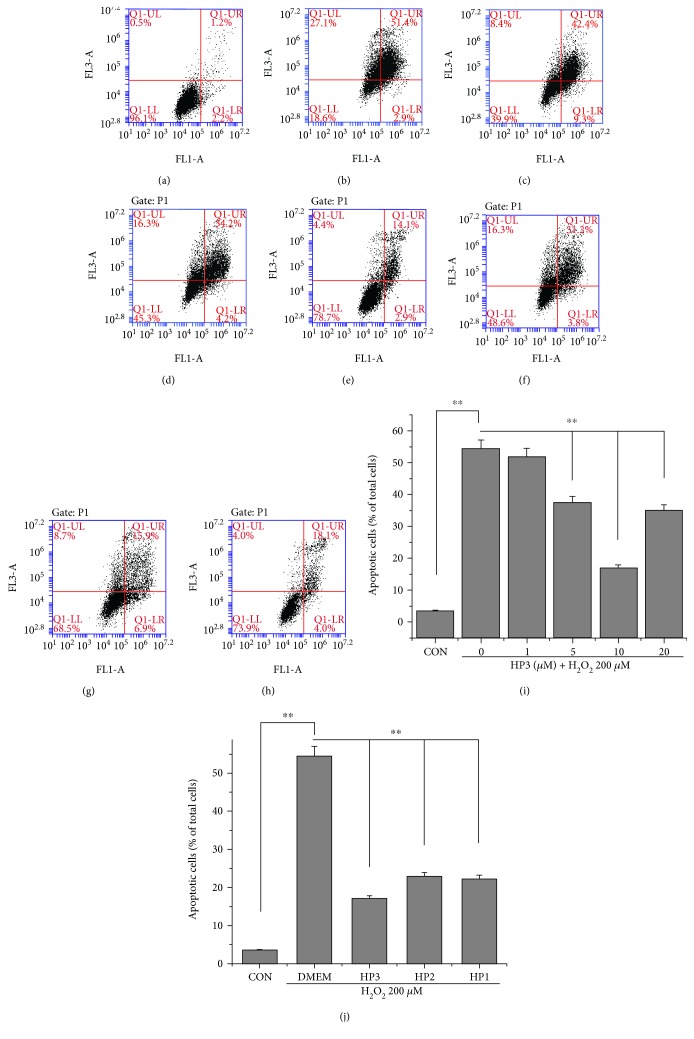
Effects of HP1, HP2, and HP3 on H_2_O_2_-induced apoptosis in EA.hy926 cells as measured by flow cytometry. (a) Control group; (b) 200 *μ*M H_2_O_2_ treatment group; (c–f) 1, 5, 10, and 20 *μ*M HP3, respectively, followed by the treatment of 200 *μ*M H_2_O_2_; (g-h) 10 *μ*M HP2 and HP1, respectively, followed by the treatment of 200 *μ*M H_2_O_2_. (i) HP3 decreased the percent of apoptotic cells induced by 200 *μ*M H_2_O_2_ in a dose-dependent manner below 10 *μ*M. (j) HP1, HP2, and HP3 protected EA.hy926 cells against H_2_O_2_-induced apoptosis. ^∗∗^*P* < 0.01.

To determine whether the cytoprotective actions of halophenols are also involved in the inhibition of cell apoptosis, the effects of HP3, HP2, and HP1 on apoptosis following H_2_O_2_-induced injury in EA.hy926 cells were also evaluated by flow cytometric analysis in parallel with the MTT assay. As shown in Figure 3, the percentage of apoptotic cells (upper right) increased from 3.4% in the control group to 54.3% in the H_2_O_2_-only treated group. However, pretreatment of the cells with 1, 5, 10, and 20 *μ*M HP3 significantly decreased rates of apoptosis induced by H_2_O_2_ to 51.7%, 37.4%, 17%, and 35%, respectively (Figure 3(i)). Pretreatment of cells with 10 *μ*M HP2 and HP1 also significantly attenuated cell apoptosis induced by H_2_O_2_ to 22.8% and 22.1%, respectively (Figure 3(j)). These results suggest that HP1, HP2, and HP3 protect EA.hy926 cells against H_2_O_2_-induced apoptosis.

### 3.3. Halophenols Inhibit H_2_O_2_-Induced Production of ROS and TNF-*α* in EA.hy926 Cells

**Figure 4:**
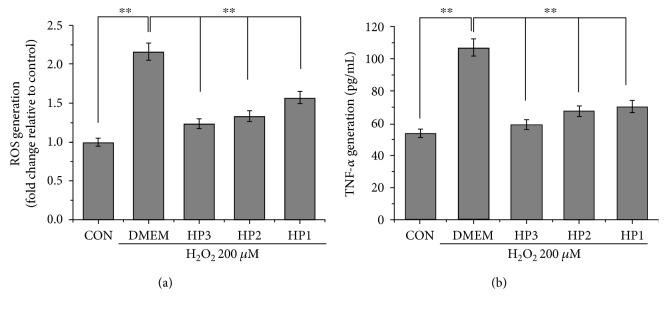
Halophenols inhibit H_2_O_2_-induced production of ROS and TNF-*α* in EA.hy926 cells. (a) HP1, HP2, and HP3 decreased the ROS release in the order of HP3 > HP2 > HP1 at 10 *μ*M. Confluent cells in 6-well plates were pretreated with 10 *μ*M compound for 6 h, followed by exposure to 200 *μ*M H_2_O_2_ for 30 min. ROS generation of HP1, HP2, and HP3 was determined by measuring DCFH-DA by flow cytometric analysis. (b) HP1, HP2, and HP3 inhibited the TNF-*α* secretion in the order of HP3 > HP2 > HP1 at 10 *μ*M. Confluent cells in 24-well plates were pretreated with compound for 6 h, subsequently incubated with 200 *μ*M H_2_O_2_ for 30 min. TNF-*α* released into the medium was detected using an ELISA kit according to the manufacturer's instructions. ^∗∗^*P* < 0.01.

H_2_O_2_ causes cell damage by promoting the synthesis of several inflammatory mediators, including ROS and TNF-*α*. Therefore, we next examined whether the halophenols were capable of reducing ROS generation and TNF-*α* secretion in H_2_O_2_-induced EA.hy926 cells. As shown in Figure 4, intracellular ROS formation and TNF-*α* levels in the cell supernatant were significantly elevated after exposure to 200 *μ*M H_2_O_2_. However, halophenol treatments efficiently reduced ROS and TNF-*α* levels in H_2_O_2_-treated cells, with the strongest effect exhibited by HP3, followed by HP2 and HP1, in accordance with their cytoprotective activities. These findings indicate that halophenols inhibit the H_2_O_2_-induced production of ROS and TNF-*α* in EA.hy926 cells.

### 3.4. HO-1 Is Involved in the Cytoprotective Actions of Halophenols

**Figure 5:**
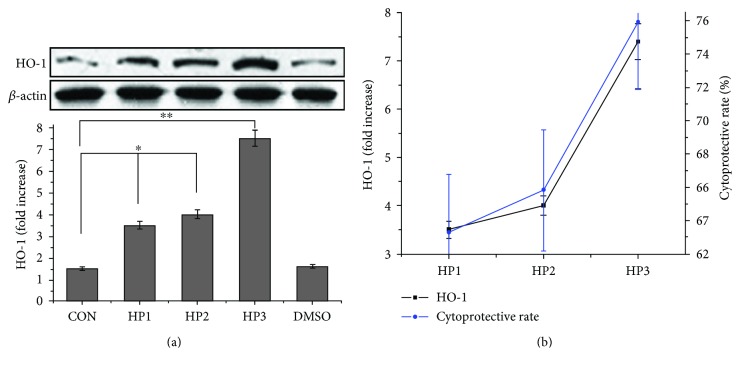
HP1, HP2, and HP3 activate the HO-1 expression in EA.hy926 cells. (a) The HO-1 induction actions of HP1, HP2, and HP3 at 10 *μ*M. The induction action for HP3 ranked first, followed by HP2 and HP1 in the order. (b) The correlation between the HO-1 relative expression and cytoprotective rates of HP1, HP2, and HP3. ^∗^*P* < 0.05 and ^∗∗^*P* < 0.01.

As HO-1 performs a vital function in the cellular defense against oxidative stress, we assessed whether the halophenols could induce HO-1 protein expression in relation to their cytoprotective actions. All three halophenols enhanced HO-1 protein expression at a concentration of 10 *μ*M, with HP3 inducing the highest HO-1 expression, followed by HP2 and HP1 (Figure 5(a)). Interestingly, this order was consistent with their cytoprotective actions (Figure 5(b)). This demonstrates that there is likely a strong correlation between HO-1 activation and the cytoprotective actions of halophenols.

### 3.5. ZnPP, an Inhibitor of HO-1, Suppresses the Cytoprotective Effects of Halophenols against H_2_O_2_-Induced Cell Damage

**Figure 6:**
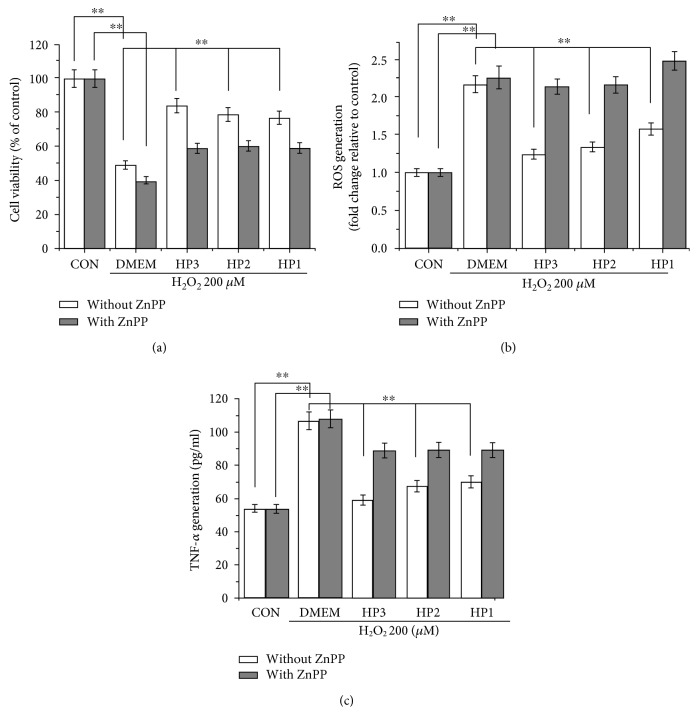
ZnPP suppresses the cytoprotective effects of halophenols against H_2_O_2_-induced EA.hy926 cell damage. (a) ZnPP suppresses the cytoprotective effects of 10 *μ*M HP1, HP2, and HP3 on H_2_O_2_-induced cell damage. (b) ZnPP reduces the inhibitory effects of 10 *μ*M HP1, HP2, and HP3 on the ROS production. (c) ZnPP decreases the inhibitory effects of 10 *μ*M HP1, HP2, and HP3 on the TNF-*α* secretion. ^∗∗^*P* < 0.01.

ZnPP, a specific inhibitor of HO-1, was used to further investigate whether HO-1 induction participated in the protective effects of halophenols against H_2_O_2_-induced cytotoxicity of EA.hy926 cells. Cells were treated with 10 *μ*M ZnPP and then incubated with 10 *μ*M halophenol for 6 h prior to treatment with 200 *μ*M H_2_O_2_ for an additional 6 h [39]. MTT results demonstrated that the protective effects of the three halophenols against H_2_O_2_-induced cytotoxicity were reversed by the addition of ZnPP (Figure 6(a)). Analysis of intracellular ROS generation showed that ZnPP suppressed the ROS-scavenging activity of halophenols, and high TNF-*α* levels were restored in cell supernatants after ZnPP treatment in the presence of halophenols and H_2_O_2_ (Figures 6(b) and 6(c)). This suggests that the induction of HO-1 protein expression participates in the actions of halophenols against H_2_O_2_-induced cell death and production of ROS and TNF-*α* in EA.hy926 cells. Taken together, the evidence indicates that the inhibition of ROS and TNF-*α* generation by halophenols is required for HO-1 activation. Moreover, HO-1 appears to play a key role in the cytoprotective actions of halophenols by inhibiting ROS and TNF-*α* generation.

### 3.6. HP3 Increases HO-1 Expression by Activating Nrf2

**Figure 7:**
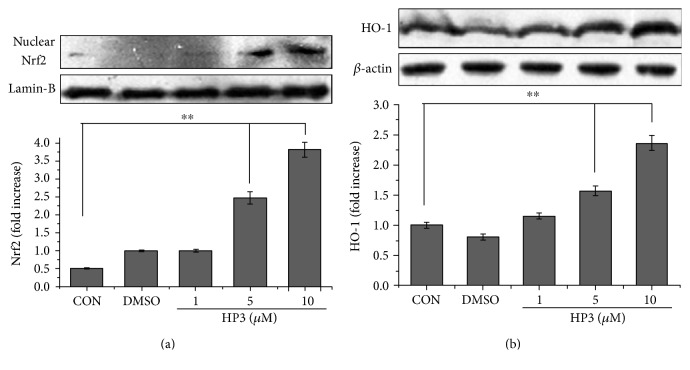
HP3 increases HO-1 expression by activating Nrf2 in EA.hy926 cells. (a) Induction of HP3 on nuclear Nrf2 expression. Cells were treated with HP3 at the indicated concentrations for 6 h. Nuclear extracts were prepared and 40 *μ*g protein samples were subjected to western blot assay by using an anti-Nrf2 antibody or an anti-Lamin B (a nuclear protein marker) antibody. HP3 increased nuclear Nrf2 protein levels in a dose-dependent manner below 10 *μ*M. (b) HP3 induced HO-1 expression in a concentration-dependent manner below 10 *μ*M. ^∗∗^*P* < 0.01.

The nuclear translocation of activated Nrf2 is an important upstream contributor to the transcription of HO-1 [32]. Therefore, we selected HP3 to evaluate the role of Nrf2 in halophenol cytoprotection. To determine whether HP3 stimulates Nrf2 translocation in EA.hy926 cells, cells were treated with different concentrations of HP3 for 6 h, and the nuclear fractions were extracted for preparation of nuclear proteins. Nrf2 proteins in the cellular nuclear compartments were detected by western blotting. As shown in Figure 7(a), HP3 enhanced nuclear Nrf2 expression in a concentration-dependent manner. Furthermore, consistent cytosolic protein expression of HO-1 was observed after HP3 treatment (Figure 7(b)). These results suggest that HP3-induced expression of HO-1 is mediated by activation of Nrf2 in EA.hy926 cells.

### 3.7. Involvement of Erk1/2 and Akt Kinase Pathways in HP3-Induced HO-1 Expression

**Figure 8:**
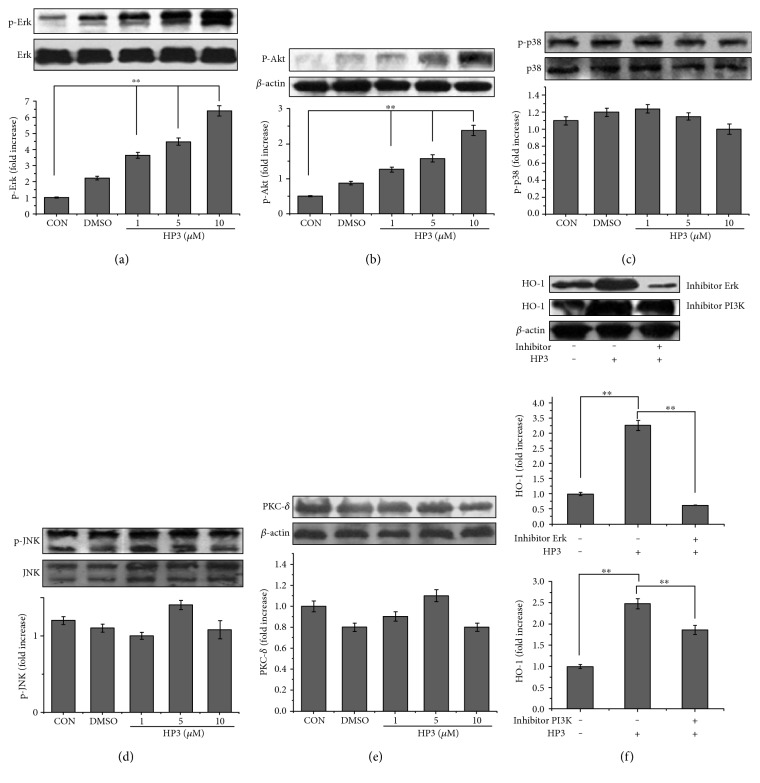
Mediation of HP3 on MAPK, PI3K/Akt, and PKC signaling pathways. (a) HP3 induced Erk1/2 phosphorylation in a concentration-dependent manner. (b) HP3 induced Akt phosphorylation in a concentration-dependent manner. (c) HP3 had no influence on p38 phosphorylation. (d) HP3 had no influence on JNK phosphorylation. (e) HP3 had no influence on PKC-*δ* protein expression. (f) Influences of the inhibitors PD98059 and Wortmannin on HO-1 expression. The uses of inhibitors significantly reduced HO-1 expression compared to those of treatment with HP3 alone. ^∗∗^*P* < 0.01.

MAPK cascades have been principally implicated in HO-1 activation. Many studies have suggested that the MAPK, PI3K/Akt, and PKC cascade pathways are involved in Nrf2 activation and translocation for the synthesis of highly specialized proteins, including HO-1. Subsequent experiments were designed to determine the possible roles of these pathways in HP3-induced HO-1 expression. As shown in Figures 8(a) and 8(b), HP3 treatment caused a concentration-dependent increase in the phosphorylation of Erk1/2 and Akt but had no effect on the expression of phosphorylated p38 (Figure 8(c)), phosphorylated JNK (Figure 8(d)), or PKC-*δ* (Figure 8(e)). In addition, when PD98059 (an inhibitor of Erk1/2) or Wortmannin (an inhibitor of PI3K) were added, HO-1 protein expression was significantly reduced compared to that in cells treated with HP3 alone (Figure 8(f)), confirming that the Erk1/2 and PI3K/Akt pathways participate in HP3-mediated HO-1 activation.

## 4. Discussion

The vascular endothelium is the major barrier of the cardiovascular system fighting against oxidative injury and inflammation, which is involved in the development of cardiovascular diseases including atherosclerosis, myocardial ischemia reperfusion, and hypertension [39]. The overproduction of ROS and TNF-*α* results from this oxidative stress [40, 41]. ROS production is tightly controlled by endogenous antioxidant systems; however, when endogenous antioxidants are overwhelmed by overproduction of ROS, oxidative stress occurs, resulting in inflammation and cellular damage [42]. This is characterized by the overproduction of inflammatory mediators, such as TNF-*α* and interleukin-6 (IL-6) [43].

In recent years, many novel halophenols have been reported for their wide spectra of bioactivities, including antioxidant, antithrombotic, antimicrobial, anti-inflammatory, enzyme inhibitory, cytotoxic, and feeding-deterrent activities [3–7, 44]. In our previous research, a series of benzophenone, benzylbenzene, and furan-2-yl(phenyl)methanone halophenols were prepared [8, 9]. Among these, three benzophenone halophenols, HP1, HP2, and HP3, exhibited strong antioxidant properties. In particular, HP3 also showed significant antiatherosclerotic and protective effects against myocardial ischemia-reperfusion injury in rats [8, 13, 14]. The attenuation of oxidative stress has been suggested to contribute to the cardiovascular benefits of active halophenols, implying that halophenols have potential value in the treatment of oxidative stress-associated cardiovascular diseases. This information prompted us to investigate whether there were significant correlations between the cytoprotective actions of halophenols and their antioxidant capabilities.

Although primary endothelial cells are widely considered a superior model for studying of endothelial cell biology than other cell lines, they may require complex growth medium and undergo phenotypic change with passages. Nowadays, EA.Hy926 cell line generated by the fusion of HUVECs with the human lung carcinoma cell line A549 is also widely used for researches of vascular endothelial protective activities of chemical substances in vitro due to its expression of some factors such as VIII-related antigen (VIIIR: Ag) [45, 46], Weibel-Palade bodies [47], Von Willebrand factor [45], *α*-granule membrane protein (GMP-140) [48], prostacyclin [49], platelet activating factor [50], tissue plasminogen activator, plasminogen activator inhibitor type I [51], thrombomodulin [52], vitronectin receptor [53], and modified low-density lipoproteins [52], which are similar to those expressed by primary endothelial cells. Zhao et al. employed EA.Hy926 cells to establish H_2_O_2_-mediated oxidative injury model for evaluation of the protective effects of pranlukast, which was associated with the inhibition of ROS-mediated collapse of mitochondrial membrane potential [54]. Wang et al. used EA.hy926 cells exposed to H_2_O_2_ to investigate the protective role of 7,8-dihydroxyflavone, which acted by inhibiting cell apoptosis, inflammatory factor releasing, and ROS level via binding to TrkB receptor [55]. Tian et al. synthesized quercetin 7-O-sialic acid and evaluated its protective effects against H_2_O_2_ or oxidized low-density lipoprotein-induced oxidative damage on EA.hy926 cells, which was related to the reduction of ROS generation [56].

In the present study, we established H_2_O_2_-induced oxidative stress injury of EA.Hy926 cells to explore the protective effects of halophenols against endothelial damage. To select a suitable concentration of H_2_O_2_ exposure, we treated cells with 50–600 *μ*M H_2_O_2_ for 6 h and measured the cell survival. The results showed that exposure of EA.hy926 cells to 200 *μ*M H_2_O_2_ for 6 h caused a reduction of cell survival in a modest but readily detectable manner. There were reported that the concentration of H_2_O_2_ frequently used to induce oxidative stress was ranging from 10 to 1000 *μ*M [57–60]. Fan et al. treated HUVECs with 200 *μ*M H_2_O_2_ to induce oxidative stress and revealed that the protective effects of *Panax notoginseng* saponins and Ginsenoside Rb1 on endothelial cells were associated with the activation of Nrf2, which mediated the suppress of monocyte adhesion events via the inhibition of the ROS-TNF-*α*-p38-VCAM-1 pathway [61]. Qiao et al. treated HUVECs with 400 *μ*M H_2_O_2_ and demonstrated that polydatin can protect HUVECs against oxidative injury by inhibition of oxidative stress and cell apoptosis via various interactions with PKC pathway [39]. On the other hand, Lacy et al. reported that human plasma contained 1–8 *μ*M H_2_O_2_ (with an average of ~3 *μ*M) under physiological condition by comparing the plasma hydrogen peroxide production of normotensive and hypertensive members [62]. Hyslop et al. found that H_2_O_2_ levels can attain 100–160 *μ*M during acute vascular pathology such as ischemia/reperfusion of rats [63]. High levels of vascular H_2_O_2_ were also observed in the transient and progressive vascular pathologies of wounds and hyperglycemia, respectively [58, 64]. That is, high-levels of H_2_O_2_ may be linked to some specific disease conditions where severe inflammation is evident.

In the following experiments, the dosing regimen of halophenols was considered in relation to the physiological actions and future clinical applications. Previously, we investigated the pharmacokinetics of HP3 in rats by oral administration HP3-solid dispersion (PVP-K30 as dispersing carrier), and the results showed that most plasma concentration values in 24 h after oral administration were kept around 10 *μ*M (unpublished). Additionally, MTT assay showed that a 10 *μ*M concentration of halophenols exhibited no significant cytotoxicity (Figure 2(a)). Therefore, we designated that the doses of the compounds were below 10 *μ*M in present research.

HO-1, a key antioxidant enzyme, has been recognized as a major component of the cellular defense system against oxidative stress and plays an essential role in the pathogenesis of cardiovascular diseases [44, 65–70]. Until now, it has remained unclear whether the induction of HO-1 was involved in the cytoprotective actions of halophenols. Accordingly, the effects of HP1, HP2, and HP3 on HO-1 protein expression were determined, and we further investigated the roles of associated signaling pathways in HO-1 activation. ZnPP is one of the most commonly used specific inhibitors of HO-1 and can reverse the protective effect of the drug against H_2_O_2_-induced injury at the cell level by influencing the activity and expression of HO-1. Thus, we selected the ZnPP as an inhibitor of HO-1 to investigate the relevance between the HO-1 activation and cytoprotective actions of halophenols. The ZnPP concentration used is not always consistent in the literature including 1, 5, 10, and 20 *μ*M [66, 71]. By our preliminary experiment, we found that 10 *μ*M ZnPP exhibited the most significant inhibition on the protective effects of halophenols. We thus determined 10 *μ*M as ZnPP concentration for this study. Our results showed that the active halophenols markedly induced upregulation of HO-1 (Figure 5(a)) and decreased intracellular levels of ROS and TNF-*α* (Figure 4). Conversely, inhibition of HO-1 by ZnPP significantly prevented the protective effects of halophenols against oxidative damage (Figure 6). Moreover, the magnitude of the induction of HO-1 by the three halophenols was correlated with their cytoprotective activities, with HP3 having the greatest effect, followed by HP2 and HP1 (Figure 5(b)). This confirms that the protective actions of the halophenols on vascular endothelium cells are closely associated with their antioxidant capabilities, as well as verifying that HO-1 is a key antioxidant enzyme involved in the cytoprotective actions of halophenols.

Nrf2 is a key regulator of endogenous antioxidant defense, and Nrf2-dependent phase II enzymes play a pivotal role in preventing oxidative stress, chronic inflammation, and atherosclerotic pathogenesis [44, 65–67, 72]. The anti-inflammatory effects of phase II antioxidant enzymes are associated with the modulation of ROS generation and removal. Among these proteins, HO-1 is a potent antioxidant enzyme, catalyzing the conversion of heme to the biologically active products CO and biliverdin/bilirubin, which regulate the inflammatory response by acting as potential antioxidants [73]. We found that the upregulation of HO-1 expression was accompanied by the activation of Nrf2 (Figure 7). We therefore conclude that the cytoprotective properties of halophenols against oxidative stress are mediated via the activation of the Nrf2/HO-1 pathway. Of note, Nrf2 can be modulated by MAPKs (ERK1/2, p38, and JNK), PI3k/Akt, and PKC kinases [27–32, 71, 74]. In order to assess whether these signaling pathways were involved in the protective actions of halophenols on vascular endothelial cells, we selected HP3, which had the highest cytoprotective activity, and investigated its effect on the MAPK/Nrf2, PI3k/Akt/Nrf2, and PKC/Nrf2 signaling pathways. Western blotting results indicated that HP3 activated the phosphorylation of Erk1/2 and Akt (Figures 8(a) and 8(b)) and upregulated Nrf2-dependent HO-1 expression (Figure 7(a)) in a dose-dependent manner, but HP3 had no obvious effect on the phosphorylation of p38 or JNK or on the expression of PKC-*δ* (Figures 8(c), 8(d), and 8(e)). In addition, HO-1 was suppressed by blocking Erk1/2 or PI3K with the specific inhibitors PD98059 or Wortmannin (Figure 8(f)). These findings suggest that HP3 triggers the phosphorylation of Erk1/2 and PI3K/Akt, mediating Nrf2 transcription and activation to induce HO-1 expression, and thereby inhibiting ROS and TNF-*α* generation for enacting its protective effect against oxidative stress injury.

The involvement of the Erk1/2 and PI3K/Akt kinase pathways and their antiapoptotic effects in HP3-mediated HO-1 activation strongly supports the role of HO-1 in directly quenching oxidative stress, normalizing intracellular redox balance, and enhancing cellular survival. This heme-degrading protein also activates alternate cell survival pathways that inhibit apoptosis.

**Figure 9:**
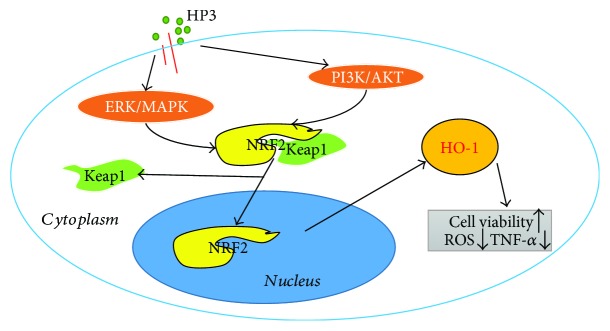
Diagram of the proposed molecular mechanism of HP3 in the induction of HO-1 to protect against oxidative stress injury in EA.hy926 cells.

In conclusion, the antiapoptotic, antioxidant, and anti-inflammatory abilities of halophenols contribute to their cytoprotective actions against H_2_O_2_-induced injury in EA.hy926 cells. In this process, HO-1 acts as a key inducible enzyme. The protective actions of active halophenols are mediated by the upregulation of Nrf2-dependent HO-1 expression and reductions in ROS and TNF-*α* generation via activation of the Erk1/2 and PI3K/Akt signaling pathways in EA.hy926 cells (Figure 9). Our findings reveal that HO-1 may be an important potential target for further structural optimization of active halophenol derivatives in the development of treatments for oxidative stress-associated cardiovascular diseases.
